# Isolation and characterization of non-rhizobial bacteria and arbuscular mycorrhizal fungi from legumes

**DOI:** 10.1186/s12866-024-03591-z

**Published:** 2024-11-06

**Authors:** Mohamed Hemida Abd-Alla, Nivien A. Nafady, Amany A. Hassan, Shymaa R. Bashandy

**Affiliations:** 1 Botany and Microbiology Department, Faculty of Science, Assiut University, Assiut, 71516 Egypt; 2 Botany and Microbiology Department, Faculty of Science, New Valley University, El-Kharga, 72511 Egypt

**Keywords:** Endophytic bacteria, 16S rRNA, Plant growth promotion, AMF, Isolation frequency

## Abstract

This study investigates non-rhizobial endophytic bacteria in the root nodules of chickpea (*Cicer arietinum* L), faba bean (*Vicia faba*), and cowpea (*Vigna unguiculata* L. Walp), as well as arbuscular mycorrhizal fungi in the rhizospheric soil of chickpea and faba bean. Out of the 34 endophytic bacterial populations examined, 31 strains were identified as non-rhizobial based on nodulation tests. All strains were assessed for their plant growth-promoting (PGP) activities in vitro. The results revealed that most isolates exhibited multiple PGP activities, such as nitrogen fixation, indole-3-acetic acid (IAA) and ammonia (NH_3_) production, phosphate solubilization, and exopolysaccharide production. The most effective PGP bacteria were selected for 16S rRNA analysis. Additionally, a total of 36 species of native arbuscular mycorrhizal fungi (AMF) were identified. *Acaulospora* (100%) and *Scutellospora* (91.66%) were the most prevalent genera in *Cicer arietinum* L. and *Vicia faba* L. plants, respectively. *Acaulospora* also exhibited the highest spore density and relative abundance in both plants. Moreover, the root colonization of *Cicer arietinum* L. and *Vicia faba* L. plants by hyphae, vesicles, and arbuscules (HVA) was significant. The findings of this study provide valuable insights into non-rhizobial endophytic bacteria associated with legume root nodules and the diversity of AMF. These organisms have great potential for PGP and can be manipulated by co-inoculation with rhizobia to enhance their biofertilizer effectiveness. This manipulation is crucial for promoting sustainable agriculture, improving crop growth, and advancing biofertilizer technology.

## Introduction

Plant growth-promoting bacteria (PGPB) are a diverse group of bacteria that provide beneficial effects to their hosts and are present in the rhizosphere, at root surfaces, and in association with roots. Endophytic bacteria are a specific group of PGPB [1, 2]. Numerous studies have shown that symbiotic and non-symbiotic bacteria have been isolated from the root nodules of various legumes [3, 4]. Non-symbiotic endophytes live within nodules but do not cause visible harm to their hosts. Endophytic bacteria reside within apoplastic spaces inside the plant or occupy intracellular spaces [5]. Recently, endophytic bacteria have been recognized as a potential group of PGPB [6]. Many PGPBs directly or indirectly stimulate plant growth and improve plant quality. They can be used as biofertilizers [7], biopesticides [8], and bioremediation agents [9]. PGPB also have advantages in increasing plant tolerance to various abiotic stresses [10]. Indirectly, they help control pathogenic microorganisms such as fungal pathogens *Rhizoctonia solani, Sclerotinia sclerotiorum, Verticillium dahliae,* and *Phytophthora cactorum*, as well as bacterial pathogens *Erwinia carotovora, Streptomyces scabies,* and *Xanthomonas campestris* [11]. Genera of Rhizobia, *Azospirillum, Bacillus, Brevibacillus, Acetobacter, Nitrobacter, Bradyrhizobium, Mesorhizobium, Azotobacter, Enterobacter, Serratia, Paenibacillus,* and *Stenotrophomonas* have been reported to enhance plant growth [12–14]. They directly promote plant growth by producing numerous phytohormones such as indole acetic acid (IAA), gibberellic acid, ammonia, cytokinins, and ethylene [15, 16]. Generally, the quantities of IAA produced by plants are not sufficient for their growth and development [17]. However, PGPB are capable of synthesizing IAA [18]. Endophytic bacteria promote plant growth by mobilizing different micro- and macro-nutrient contents and facilitating the uptake of these nutrients from the soil to the plants [19]. Phosphate-solubilizing bacteria (PSB) have a significant effect on phosphorus solubilization [20, 21]. Nowadays, PSB are considered an effective method for reducing the use of phosphorus fertilizers in different crops [22, 23]. Ammonia production is another PGP trait that can be used by plants as a source of nitrogen [24]. Endophytic bacteria have been isolated from legume plants such as alfalfa (*Medicago sativa*) [25], red clover (*Trifolium pratense*) [26], cowpea (*Vigna unguiculata*) [27], chickpea (*Cicer arietinum*) [28], faba bean (*Vicia faba*) [29], and soybean (*Glycine max*) [30]. To achieve maximum growth promotion in plants, it is important to develop efficient strains that produce multiple plant growth-promoting activities.

Mycologists and plant scientists are typically well-versed in the function of arbuscular mycorrhizal fungi [AMF] and their consequences for nutrient cycling and plant productivity [31]. The discovery of arbuscular mycorrhiza was made over one hundred years ago, and these associations exist in over 80% of plants [32]. AMF are ubiquitous obligate mycobionts that form a symbiotic association between host plants and certain groups of fungi in the root system [33]. AMF improve water and nutrient uptake, particularly phosphorus, in plants. They also increase the uptake of macronutrients such as nitrogen, potassium, and magnesium [34], as well as some micronutrients [35]. Additionally, AMF production of growth hormones, proteins, lipids, and sugars, as well as their ability to increase plant survival and establishment, contribute to various nutritional, biochemical, physiological, and morphological responses in plants [36]. Spore quantification has proven useful in evaluating the level and diversity of mycorrhizas because spores are highly resistant to adverse conditions [37]. It has been observed that the soil mycelium of AMF is coated with a mucilaginous substance that causes soil particles to adhere together [38]. The population variation of these fungi and their symbiosis with plant roots is influenced by both soil properties and host plants [39].

The main goal of this study was to isolate and characterize endophytic non-rhizobial bacteria found in the root nodules of chickpea (*Cicer arietinum* L), faba bean (*Vicia faba* L), and cowpea (*Vigna unguiculata* L Walp) plants. The study also aimed to assess the nitrogen-fixing abilities of these bacteria, as well as their production of growth substances such as indole acetic acid, ammonia, phosphate solubilization, and exopolysaccharides (EPS). This comprehensive assessment aimed to explore the potential of these microorganisms in enhancing plant growth and development. Additionally, the study aimed to isolate and identify native arbuscular mycorrhizal fungi (AMF) in the rhizosphere of chickpea (*Cicer arietinum* L) and faba bean (*Vicia faba* L.) plants in selected sites within the Assiut Governorate. The researchers sought to evaluate the distribution, spore density, isolation frequency, relative abundance, and colonization patterns of these AMF species to gain insights into their diversity and prevalence in the region, contributing to the overall understanding of their ecological importance and potential applications in sustainable agriculture. Ultimately, the research aimed to gather valuable information to enhance our understanding of the diversity and potential of these microorganisms. By identifying promising endophytic bacteria and native AMF, this study laid the foundation for their potential use as biofertilizer inoculants in modern agricultural practices. The development of such biofertilizer technologies holds great promise for improving crop productivity, reducing reliance on synthetic fertilizers, and promoting environmentally friendly and sustainable farming systems.

## Materials and methods

### Isolation of bacteria

Root nodules were sampled from chickpea (*Cicer arietinum* L), faba bean (*Vicia faba*), and cowpea (*Vigna unguiculata* L*. Walp)* plants grown in Assiut Governorate. The specific locations were El Qossia (53.70′30°48 East and 15.86′27°26 North), Manfalout (17.93′31°00 East and 38.55′27°14 North), Arab Elmadabegh (03.03′31°09 East and 28.96′27°10 North), Al Hawatkah (08.17′31°01 East and 19.53′27°16 North), and the Botanical Garden of the Faculty of Science (13.75′31°10 East and 25.20′27°11 North). The sampling took place during the growth season in January 2017. The nodules were surface sterilized in 95% ethanol for 15 s, then placed in 2% sodium hypochlorite for 3 min, and finally rinsed with sterile water five times. After crushing the nodules, the suspension was streaked on yeast extract mannitol agar medium (YEMA) supplemented with 10 g/L congo red, 10 g mannitol, 0.5 g yeast extract, 0.5 g K_2_HPO_4_, 0.2 g MgSO_4_, 0.1 g NaCl, 18 g agar, and 0.025 g Congo red. The pH of the medium was adjusted to 7 [40]. Colonies appeared after 24–72 h of incubation at 28 °C in aerobic conditions. Pure cultures were stored at -80 °C in 80% glycerol.

Nodulation tests were conducted on *Vicia faba* L. (Giza 402), *Cicer arietinum* L. (Giza 531), and *Vigna unguiculata* (L.) Walp. (Cream 7) obtained from the Agriculture Research Center (ARC) in Cairo, Egypt. The nodulation tests were conducted following the procedure described in a previous study [41].

### Conventional biochemical and physiological characterization of the bacterial isolates

Bacteriological characteristics such as Gram stain, utilization of sole carbon and nitrogen sources, resistance to antibiotics, tolerance to dyes for growth, catalase test, H_2_S production, urea hydrolysis, indole test, Voges-Proskauer test, gelatin hydrolysis, nitrate test, citrate test, and starch hydrolysis were determined. The isolates were examined using the methods described in Bergey’s Manual [42].

### Screening of bacterial isolates for their plant growth promoting (PGP) traits in vitro

#### Acetylene reduction assay (Nitrogenase activity)

The non-rhizobial isolates were grown in Burk's N-free medium, which contained glucose (10 g/L), KH_2_PO_4_ (0.41 g/L), K_2_HPO_4_ (0.52 g/L), Na_2_SO_4_ (0.05 g/L), CaCl_2_ (0.2 g/L), MgSO_4_·7H_2_O (0.1 g/L), FeSO_4_·7H2O (0.005 g/L), and Na_2_MoO_4_·2H_2_O (0.0025 g/L). The bacterial isolates were then incubated at 37 °C for 48 h. After incubation, the nitrogenase activity (N2ase) of the isolates was assessed using the acetylene reduction assay (ARA) in a closed system [41]. Nitrogen gas was introduced into the bacterial cultures in 15 ml sterile serum bottles (25 ml) sealed with rubber septa using a sterile hypodermic needle. After removing 10% of the air from the headspace of each bottle, it was replaced with acetylene gas. The bottles were then incubated for one hour at 37 °C. The ethylene generated was determined using a Thermo Scientific Trace GC Ultra gas chromatograph equipped with a manual injector, injector loop, sample splitter, and flame ionization detector (FID). The analysis was performed using a CP-Pora Bond U fused silica plot capillary column (25 m × 0.32 mm, 7 µm) connected to the FID detector.

#### Test of IAA production ability

The non-rhizobial isolates were tested for indole-3-acetic acid (IAA) production, as reported [43]. Bacterial cultures were grown in a nutrient broth medium supplemented with tryptophan (2 mg ml^−1^) at 28 °C for 5 days. The cultures were then centrifuged, and 2 ml of the supernatant was mixed with 2 ml of a reagent consisting of 4.5 g of FeCl_3_ per liter in 10.8 M H_2_SO_4_. The mixture was incubated at room temperature for 25 min, and the absorbance of the pink color that developed was read at 530 nm using a spectrophotometer (Unicam, Helios γ). A calibration curve was created using pure IAA.

#### Detection of phosphate solubilizing ability

The isolates were tested by plate assay using Pikovskaya phosphate medium (PVK) [44]. The composition of the medium was as follows: Glucose 10 g, CaHPO_4_ 5 g, (NH_4_)_2_SO_4_ 0.5 g, NaCl 0.2 g, MgSO_4_.7H_2_O 0.1 g, KCl 0.2 g, yeast extract 0.5 g, MnSO_4_.H_2_O 0.002 g and FeSO_4_.7H_2_O 0.002 g. The medium was made up to one liter with distilled water and adjusted to pH 6.8. The tested isolates were stabbed on the plate in three replicates using the sterilized loop. After 5 days of incubation at 28 °C, the clear zone appeared around the colony indicating the phosphate solubilization ability of the isolates.

#### Ammonia production test

The non-rhizobial isolates were tested to produce ammonia in peptone water. Test tubes containing 10 ml of peptone water were inoculated and incubated for 48–72 h at 28 ± 2 °C. Ammonia production was tested by adding 0.5 ml of Nessler's reagent. The development of a yellow-to-brown color indicated a positive result for ammonia production [43].

#### Exopolysaccharide production test

The non-rhizobial isolates were screened for their capacity to produce exopolysaccharide (EPS). The tested isolates were grown on YEMA medium at 28 °C for 48 h, and the colonies that formed gummy/mucoid or exudates were chosen for further studies. EPS production in liquid medium was carried out by separately growing 6 × 10^6^ cells/mL of bacterial isolates in yeast mannitol broth (YEMB) medium at 30 °C for 3 days with constant shaking at 120 rpm. After incubation, the culture media were observed for viscosity. An increase in the viscosity of the culture broth indicated a positive result for EPS production [45].

#### 16S rRNA gene amplification and sequencing

The bacterial isolates were sent to SolGent Company (Solgent Co., Ltd, Bio Industry Development Site, 63–10 Hwaan-Dong, Yuseong-Gu, Daejeon, South Korea) for analysis of 16S rRNA gene sequences. The sequence reads were edited and assembled using BioEdit version 7.0.4 (http://www.mbio.ncsu.edu/BioEdit/bioedit.html) and clustal W version 1.83 (http://clustalw.ddbj.nig.ac.jp/top-e.html). BLAST searches were performed using the NCBI server at http://www.ncbi.nlm.nih.gov/blast/Blast.cgi. Phylogenetic trees based on the 16S rRNA gene sequences were constructed using sequences from different standard bacterial strains obtained from Genbank.

## Nucleotide sequence accession number

The nucleotide sequence of bacterial isolates (C1 to C5), Vf1, Vf2, Vf3, Vu1, and Vu2 isolated from chickpea *(Cicer arietinum L), *faba bean* (Vicia faba), and *cowpea* (Vigna unguiculata L. Walp)* plants, were deposited in the GenBank nucleotide sequence database under accession number KY515467.1(https://www.ncbi.nlm.nih.gov/nucleotide/KY515467.1?report=genbank&log$=nuclalign&blast_rank=1&RID=M1JC3T6T016), MH398502.1(https://www.ncbi.nlm.nih.gov/nucleotide/MH398502.1?report=genbank&log$=nuclalign&blast_rank=1&RID=M1JG3D30013) MH400058.1(https://www.ncbi.nlm.nih.gov/nucleotide/MH400058.1?report=genbank&log$=nuclalign&blast_rank=1&RID=M1JM6GKR013) MH398500.1(https://www.ncbi.nlm.nih.gov/nuccore/MH398500.1?report=GenBank), MG515188.1(https://www.ncbi.nlm.nih.gov/nucleotide/MG515188.1?report=genbank&log$=nuclalign&blast_rank=1&RID=M1K872J8016), MH398516.1(https://www.ncbi.nlm.nih.gov/nucleotide/MH398516.1?report=genbank&log$=nucltop&blast_rank=3&RID=M1KRY82N01N), MH398503.1(https://www.ncbi.nlm.nih.gov/nuccore/MH398503.1?report=GenBank), MH398497.1(https://www.ncbi.nlm.nih.gov/nuccore/MH398497.1?report=GenBank), and KY515468.1(https://www.ncbi.nlm.nih.gov/nuccore/KY515468.1?report=GenBank), respectively.

### Isolation and estimation of AM fungal spore density

Mycorrhizal spores were isolated from the rhizospheric soil of *Cicer arietinum* L. and *Vicia faba* L. plants grown in Assiut Governorate, including El Qossia, Manfalout, Al Hawatkah, and the Botanical Garden of the Faculty of Science. The soil was cleaned by removing leaf litter and other debris, and the AM fungal spores were isolated using the Wet Sieving and decanting method [46]. Spores were recovered by filtering the sieved fraction onto a filter paper and then spread over a large petri dish (13.5 cm) for counting under a dissecting microscope. The intact spores were counted and recorded as the total per sample in 100 g of soil as AM fungal spore density. Slides were prepared to identify the AM fungi, and the spores were mounted on glass slides in polyvinyl-lactic acid and carefully crushed under a compound microscope (Olympus CX41) with a camera (CS30).

### Identification of AM fungal species

The spores and sporocarps were mounted in polyvinyl alcohol lactoglycerol (PVLG) and identified based on their morphology using taxonomic keys. This identification process involved considering factors such as size, color, shape, bulbous suspensor, number of wall layers, wall structure, and thickness of walls, as described in previous studies [47, 48].

### Assessment of mycorrhizal colonization in plant roots

The roots were used to estimate the proportion of root length colonized by AM fungi using the method described by [49]. The root samples were washed with tap water to remove soil particles and then preserved in a 50% ethanol solution. Fresh, rinsed roots or fixed roots were cut into short lengths (3–5 mm) and placed in a 2.5% aqueous solution of KOH (w/v), then heated in a water bath at 90 °C for 10–30 min. After treatment with KOH, the roots were rinsed in several changes of water. If the roots were not clear, they were lightened in a freshly prepared solution of alkaline H_2_O_2_. After the KOH or H_2_O_2_ treatments, roots were acidified by soaking in 1% HCl overnight. Acidified roots were stained in an acidic glycerol solution (500 mL glycerol, 450 ml H_2_O, 50 mL 1% HCl) containing 0.05% trypan blue. Roots were heated in this solution at 90 °C for 15 min. The trypan blue solution was poured off, and the roots were destained in acidic glycerol. Root segments were picked up and arranged on glass slides and examined using a light microscope (Olympus) for the presence and intensity of mycorrhizal hyphae, arbuscles, and vesicles. The percentage of AM infection was calculated using the following equation: (Total number of colonized root segments / Total number of root segments studied) × 100.

### Statistical analysis

The data underwent a one-way ANOVA using the SPSS 21.0 software program. Standard errors and means were calculated for three replicates. The means were separated by Duncan's multiple range test, and statistical significance was determined at the 5% level.

## Results

### Isolation and characterization of the bacterial isolates

Non-rhizobial isolates were successfully obtained from the root nodules of *Cicer arietinum* L*., Vicia faba* L*.,* and* Vigna unguiculata* L. Walp. The nodules were sterilized to prevent the isolation of rhizobacteria from the nodule surface. A total of 34 bacterial isolates were obtained from the root nodules of these three leguminous plants, with 19 isolates from chickpea, 7 from faba bean, and 8 from cowpea. The bacterial isolates developed colonies on yeast extract mannitol medium (YEM) plates after approximately 48–72 h. Out of the 34 isolates, 31 were unable to form nodules in their respective plant hosts even after repeated inoculation. This included 17 isolates from chickpea, 6 from faba bean, and 8 from cowpea plants.

### Conventional biochemical and physiological characterization of the bacterial isolates

Most bacterial isolates tested positive for catalase, H_2_S, and Voges-Proskauer tests. Out of the 31 endophytic bacterial isolates recovered from root nodules, 24 were gram-negative (Table 1). Only three isolates (C6, C14, and Vu6) showed positive results for urea hydrolysis. All isolates tested negative for the indole test, gelatin hydrolysis, and starch hydrolysis (Table 1). However, all isolates tested positive for the nitrate test, while four isolates (C16, C17, Vu1, and Vu6) tested positive for the citrate test (Table 1). Of the 31 endophytic bacterial isolates, 26 were able to grow well on various carbon sources such as fructose, galactose, glucose, maltose, sucrose, and mannitol. None of the isolates were able to grow on D-Alanine and L-glutamine, but all isolates were able to grow on peptone as a nitrogen source (Table 1). Most of the bacterial isolates showed resistance to 5 µg/ml ampicillin, 5 µg/ml chloramphenicol, and 1% Congo red, but they were sensitive to 100 µg/ml chloramphenicol, 100 µg/ml kanamycin, 0.1% Crystal violet, and 0.1% Methylene blue. Additional results can be found in Table 1.

**Table 1:** Biochemical tests of rhizobial and non-rhizobial bacterial isolates recovered from root nodules of *Cicer arietinum* (L.), *Vicia faba* (L.) and *Vigna unguiculata* (L.) Walp

Test	Isolate No. of *Cicer arietinum* (L.)																			Isolate No. of *Vicia faba* (L.)							Isolate No. of *Vigna unguiculata* (L.) Walp							
	**C1**	**C2**	**C3**	**C4**	**C5**	**C6**	**C7**	**C8**	**C9**	**C10**	**C11**	**C12**	**C13**	**C14**	**C15**	**C16**	**C17**	**C18**	**C19**	**Vf1**	**Vf2**	**Vf3**	**Vf4**	**Vf5**	**Vf6**	**Vf7**	**Vu1**	**Vu2**	**Vu3**	**Vu4**	**Vu5**	**Vu6**	**Vu7**	**Vu8**
**KOH test**	**-**	**-**	**-**	**-**	**-**	**-**	**-**	**-**	**-**	**-**	**-**	** + **	** + **	**-**	**-**	**-**	** + **	**-**	**-**	**-**	**-**	**-**	** + **	**-**	** + **	**-**	**-**	** + **	**-**	** + **	**-**	**-**	**-**	**-**
**Catalase test**	** + **	**-**	** + **	** + **	** + **	** + **	** + **	** + **	** + **	**-**	**-**	** + **	** + **	** + **	**-**	** + **	** + **	** + **	** + **	** + **	** + **	** + **	** + **	** + **	**-**	** + **	**-**	** + **	**-**	** + **	** + **	** + **	** + **	** + **
**H**_**2**_**S production**	** + **	**-**	** + **	** + **	** + **	** + **	** + **	** + **	**-**	** + **	** + **	** + **	** + **	** + **	** + **	**-**	** + **	** + **	** + **	** + **	** + **	** + **	** + **	**-**	** + **	** + **	** + **	** + **	** + **	** + **	**-**	**-**	** + **	** + **
**Urea hydrolysis**	**-**	**-**	**-**	**-**	**-**	** + **	**-**	**-**	**-**	**-**	**-**	**-**	**-**	** + **	**-**	**-**	**-**	**-**	**-**	**-**	**-**	**-**	**-**	**-**	**-**	**-**	**-**	**-**	**-**	**-**	**-**	** + **	**-**	**-**
**Indole test**	**-**	**-**	**-**	**-**	**-**	**-**	**-**	**-**	**-**	**-**	**-**	**-**	**-**	**-**	**-**	**-**	**-**	**-**	**-**	**-**	**-**	**-**	**-**	**-**	**-**	**-**	**-**	**-**	**-**	**-**	**-**	**-**	**-**	**-**
**Voges Proskauer**	** + **	** + **	** + **	** + **	**-**	** + **	** + **	** + **	** + **	**-**	** + **	** + **	** + **	** + **	** + **	** + **	** + **	** + **	** + **	** + **	** + **	** + **	** + **	** + **	** + **	** + **	** + **	** + **	** + **	** + **	** + **	** + **	** + **	** + **
**Gelatin hydrolysis**	**-**	**-**	**-**	**-**	**-**	**-**	**-**	**-**	**-**	**-**	**-**	**-**	**-**	**-**	**-**	**-**	**-**	**-**	**-**	**-**	**-**	**-**	**-**	**-**	**-**	**-**	**-**	**-**	**-**	**-**	**-**	**-**	**-**	**-**
**Nitrate test**	** + **	** + **	** + **	** + **	** + **	** + **	** + **	** + **	** + **	** + **	** + **	** + **	** + **	** + **	** + **	** + **	** + **	** + **	** + **	** + **	** + **	** + **	** + **	** + **	** + **	** + **	** + **	** + **	** + **	** + **	** + **	** + **	** + **	** + **
**Citrate test**	**-**	**-**	**-**	**-**	**-**	**-**	**-**	**-**	**-**	**-**	**-**	**-**	**-**	**-**	**-**	** + **	** + **	**-**	**-**	**-**	**-**	**-**	**-**	**-**	**-**	**-**	** + **	**-**	**-**	**-**	**-**	** + **	**-**	**-**
**Starch hydrolysis**	**-**	**-**	**-**	**-**	**-**	**-**	**-**	**-**	**-**	**-**	**-**	**-**	**-**	**-**	**-**	**-**	**-**	**-**	**-**	**-**	**-**	**-**	**-**	**-**	**-**	**-**	**-**	**-**	**-**	**-**	**-**	**-**	**-**	**-**
**Utilization as sole carbon source:**																																		
** Fructose**	** + **	** + **	** + **	** + **	**-**	** + **	** + **	** + **	** + **	** + **	**-**	** + **	** + **	** + **	** + **	** + **	** + **	** + **	** + **	** + **	** + **	** + **	** + **	** + **	** + **	** + **	** + **	** + **	** + **	** + **	** + **	** + **	** + **	** + **
** Galactose**	** + **	** + **	** + **	** + **	** + **	** + **	** + **	** + **	** + **	** + **	** + **	** + **	** + **	** + **	** + **	** + **	** + **	** + **	** + **	** + **	** + **	** + **	** + **	** + **	** + **	** + **	** + **	** + **	** + **	** + **	** + **	** + **	** + **	** + **
** Glucose**	** + **	** + **	** + **	** + **	** + **	** + **	** + **	** + **	** + **	** + **	** + **	** + **	** + **	** + **	** + **	** + **	** + **	** + **	** + **	** + **	** + **	** + **	** + **	** + **	** + **	** + **	** + **	** + **	** + **	** + **	** + **	** + **	** + **	** + **
** Maltose**	** + **	** + **	** + **	** + **	** + **	** + **	** + **	** + **	** + **	** + **	** + **	** + **	** + **	** + **	** + **	** + **	** + **	** + **	** + **	** + **	** + **	** + **	** + **	** + **	** + **	** + **	** + **	** + **	** + **	** + **	** + **	** + **	** + **	** + **
** Sucrose**	** + **	** + **	** + **	** + **	** + **	** + **	** + **	** + **	** + **	** + **	**-**	** + **	** + **	** + **	** + **	** + **	** + **	** + **	** + **	** + **	** + **	** + **	** + **	** + **	** + **	** + **	** + **	** + **	** + **	** + **	** + **	** + **	** + **	** + **
** Mannitol**	** + **	** + **	** + **	** + **	** + **	** + **	** + **	** + **	** + **	** + **	** + **	** + **	** + **	** + **	** + **	** + **	** + **	** + **	** + **	** + **	**-**	** + **	** + **	**-**	** + **	** + **	** + **	** + **	** + **	** + **	** + **	** + **	** + **	**-**
**Utilization as sole nitrogen source**																																		
** D-Alanine**	**-**	**-**	**-**	**-**	**-**	**-**	**-**	**-**	**-**	**-**	**-**	**-**	**-**	**-**	**-**	**-**	**-**	**-**	**-**	**-**	**-**	**-**	**-**	**-**	**-**	**-**	**-**	**-**	**-**	**-**	**-**	**-**	**-**	**-**
** L-glutamine**	**-**	**-**	**-**	**-**	**-**	**-**	**-**	**-**	**-**	**-**	**-**	**-**	**-**	**-**	**-**	**-**	**-**	**-**	**-**	**-**	**-**	**-**	**-**	**-**	**-**	**-**	**-**	**-**	**-**	**-**	**-**	**-**	**-**	**-**
** Peptone**	** + **	** + **	** + **	** + **	** + **	** + **	** + **	** + **	** + **	** + **	** + **	** + **	** + **	** + **	** + **	** + **	** + **	** + **	** + **	** + **	** + **	** + **	** + **	** + **	** + **	** + **	** + **	** + **	** + **	** + **	** + **	** + **	** + **	** + **
**Resistance to (µg ml-1)**																																		
** Ampicillin (5)**	** + **	** + **	** + **	** + **	** + **	** + **	** + **	** + **	** + **	** + **	**-**	** + **	** + **	** + **	** + **	** + **	** + **	** + **	** + **	** + **	** + **	** + **	** + **	** + **	** + **	** + **	** + **	** + **	** + **	** + **	** + **	** + **	** + **	** + **
** Chloramphenicol (5)**	** + **	** + **	** + **	** + **	** + **	** + **	** + **	** + **	** + **	** + **	** + **	** + **	** + **	** + **	** + **	** + **	** + **	** + **	** + **	** + **	** + **	** + **	** + **	** + **	** + **	** + **	** + **	** + **	** + **	** + **	** + **	** + **	** + **	** + **
** Chloramphenicol (100)**	**-**	**-**	**-**	**-**	**-**	**-**	**-**	**-**	**-**	**-**	**-**	**-**	**-**	**-**	**-**	**-**	**-**	**-**	**-**	**-**	**-**	**-**	**-**	**-**	**-**	**-**	**-**	**-**	**-**	**-**	**-**	**-**	**-**	**-**
** Kanamycin (100)**	**-**	**-**	**-**	**-**	**-**	**-**	**-**	**-**	**-**	**-**	**-**	**-**	**-**	**-**	**-**	**-**	**-**	**-**	**-**	**-**	**-**	**-**	**-**	**-**	**-**	**-**	**-**	**-**	**-**	**-**	**-**	**-**	**-**	**-**
** Congo red (1%)**	** + **	** + **	** + **	** + **	** + **	** + **	** + **	** + **	** + **	** + **	** + **	** + **	** + **	** + **	** + **	** + **	** + **	** + **	** + **	** + **	** + **	** + **	** + **	** + **	** + **	** + **	** + **	** + **	** + **	** + **	** + **	** + **	** + **	** + **
** Crystal violet (0.1%)**	**-**	**-**	**-**	**-**	**-**	**-**	**-**	**-**	**-**	**-**	**-**	**-**	**-**	**-**	**-**	**-**	**-**	**-**	**-**	**-**	**-**	**-**	**-**	**-**	**-**	**-**	**-**	**-**	**-**	**-**	**-**	**-**	**-**	**-**
** Methylene blue (0.1%)**	**-**	**-**	**-**	**-**	**-**	**-**	**-**	**-**	**-**	**-**	**-**	**-**	**-**	**-**	**-**	**-**	**-**	**-**	**-**	**-**	**-**	**-**	**-**	**-**	**-**	**-**	**-**	**-**	**-**	**-**	**-**	**-**	**-**	**-**

**( +) Growth present and (-) No growth**

### Detection of the bacterial isolates with plant growth-promotion traits

**Fig. 1:**
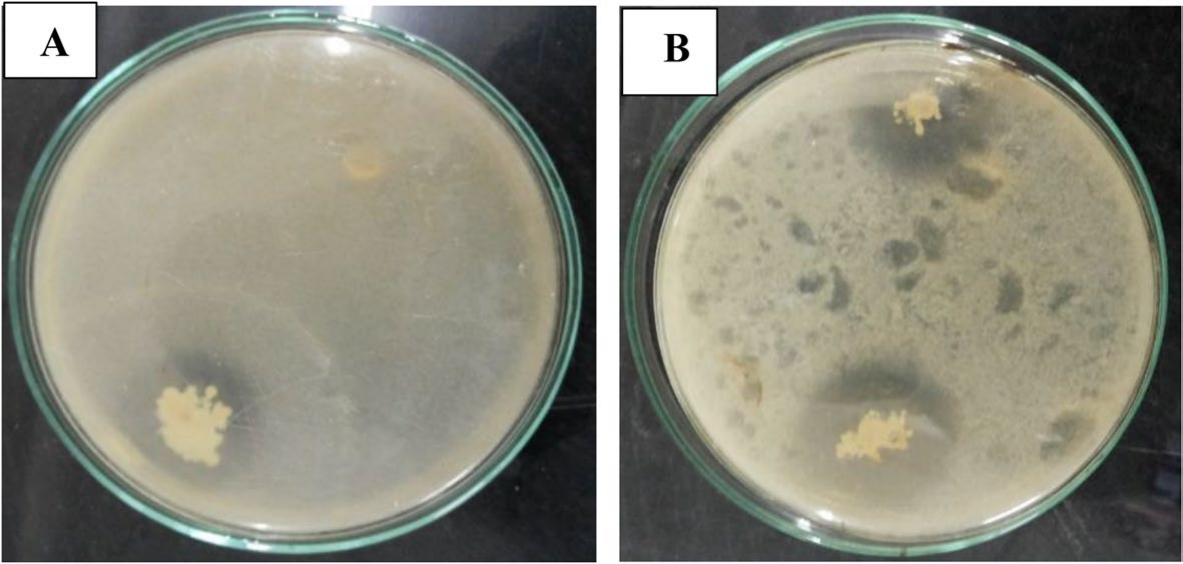
Solubilization of CaHPO_4_ by *Stenotrophomonas maltophilia* (KY515467.1) and *Bacillus cereus* (MG515188.1) on PYK Agar medium

Table 2 presents the plant growth-promoting characteristics of the bacterial isolates, including nitrogen fixation, indole-3-acetic acid (IAA) production, phosphate solubilization, ammonia production, and exopolysaccharide (EPS) production. All bacterial isolates, except for C2, C11, and C13, demonstrated the ability to fix atmospheric nitrogen. The production of IAA ranged from 2.68 to 8.94 mg ml^−1^, with the highest production observed in the isolate (C1) *Stenotrophomonas maltophilia* (KY515467.1), which was isolated from chickpea plant nodules. Two isolates, (C1) *Stenotrophomonas maltophilia* (KY515467.1) and (Vf1) *Bacillus cereus* (MG515188.1), exhibited phosphate solubilization, as indicated by the formation of a clear halo around the colony (Fig. 1). All endophytic isolates tested positive for ammonia and EPS production (Table 2).

**Table 2:** Plant growth-promoting traits of test isolates

Legume plant	Isolate no	N2ase	IAA*	Phosphate	Ammonia	EPS
			(mg/ml)	solubilization		
***Cicer arietinum*** **L**	C1	+	8.94i12	+	+	+
	C2	-	6.98g9	-	+	+
	C3	+	7.09gh910	-	+	+
	C4	+	7.46gh10	-	+	+
	C5	+	6.02ef8	-	+	+
	C6	+	8.27hi1112	-	+	+
	C7	+	3.77b3	-	+	+
	C8	+	4.46cd45	-	+	+
	C9	+	4.85d5	-	+	+
	C10	+	2.68a1	-	+	+
	C11	-	4.64d5	-	+	+
	C12	+	7.36gh10	-	+	+
	C13	-	7.07gh910	-	-	+
	C14	+	5.69E + 07	-	+	+
	C15	+	5.49E + 07	-	+	+
	C16	+	6.39fg89	-	+	+
	C17	+	3.58b3	-	+	+
***Vicia faba*** **L**	Vf1	+	4.48ab45	-	+	+
	Vf2	+	3.65a3	-	+	+
	Vf3	+	6.77d9	-	+	+
	Vf4	+	5.58c7	+	+	+
	Vf5	+	4.88bc5	-	+	+
	Vf6	+	5.69c7	-	+	+
***Vigna unguiculata*** **(L.) Walp**	Vu1	+	5.17b67	-	+	+
	Vu2	+	6.58c9	-	+	+
	Vu3	+	6.84c89	-	+	+
	Vu4	+	5.39b7	-	+	+
	Vu5	+	3.24a23	-	+	+
	Vu6	+	7.92d11	-	+	+
	Vu7	+	4.71b5	-	+	+
	Vu8	+	5.43b7	-	+	+

^*^Means with the same superscript letter among isolates within each plant cultivar and with the same superscript number among plant cultivars are not significantly different at the 0.05 level using Duncan test

### 16S rRNA gene amplification and sequencing

**Fig. 2:**
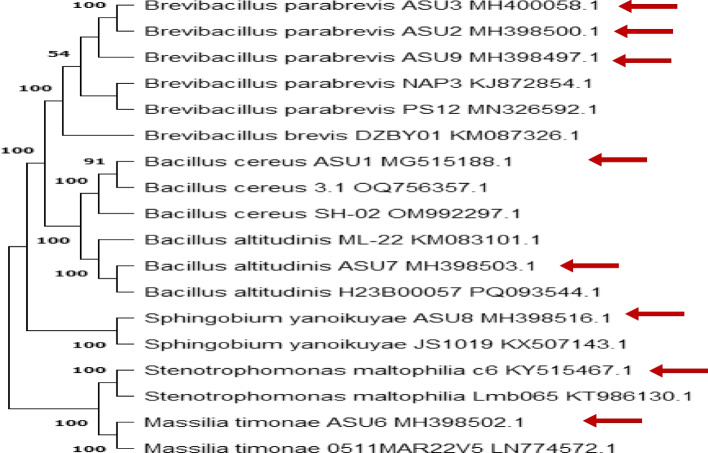
Maximum-Likelihood Phylogenetic Tree Using Tamura-Nei Model. Bootstrap values (out of 1,000) are displayed at branch points. Arrows indicate the positions of isolated bacteria. The analysis was performed in MEGA11 [104]

Previously published bacteria from the National Center for Biotechnology Information (NCBI) were used to demonstrate the relatedness of the isolates to other major groups by using the Basal Local Alignment Search Tool (BLAST) on the network site https://blast.ncbi.nlm.nih.gov/Blast. C1 was selected for further identification by conducting phylogenetic analysis of 16S rRNA gene sequences, which had a length of 1174 base pairs. This isolate had a sequence that was 97% similar to *Stenotrophomonas maltophilia* (KT986130.1). Phylogenetically, the tested isolate was closely related to the genus Stenotrophomonas, specifically the *Stenotrophomonas maltophilia* group, which belongs to the family Xanthomonadaceae, order Xanthomonadales, class Gammaproteobacteria, and the phylum Proteobacteria (Table 3; Fig. 2).

**Table 3:** Molecular identification of *Cicer arietinum* (L.),* Vicia faba* (L.), and *Vigna unguiculata* (L.) Walp. nodule endophytes by 16S rDNA sequencing

Endophyte	Species	Length (bp)	Accession number of isolated endophytes	Alignment Identity %	Accession No. of most closely related sequences, NCBI
C1	*Stenotrophomonas maltophilia*	1174	KY515467.1	97	KT986130.1
C2	*Massilia timonae*	854	MH398502.1	100	LN774572.1
C3	*Brevibacillus parabrevis*	1203	MH400058.1	95	KM087342.1
C4	*Brevibacillus parabrevis*	1203	MH398500.1	95	KX832687.1
C5	*Bacillus nealsonii*	910	MH398501.1	100	KT719591.1
Vf1	*Bacillus cereus*	887	MG515188.1	100	JQ660662.1
Vf2	*Sphingobium yanoikuyae*	1011	MH398516.1	100	KX507143.1
Vf3	*Bacillus altitudinis*	925	MH398503.1	100	MK253247.1
Vu1	*Brevibacillus parabrevis*	1185	MH398497.1	98	KJ872854.1
Vu2	*Brevibacillus parabrevis*	1228	KY515468.1	99	KM087342.1

The sequenced isolate (C2) showed a 100% match and similarity with the corresponding isolate *Massilia timonae* LN774572.1 obtained from the NCBI GenBank. The tested analyzed isolate was identified as *Massilia*, which belongs to the family Oxalobacteriaceae, order Burkholderiales, class Betaproteobacteria, and the phylum Proteobacteria. The 16S rRNA gene sequences were obtained from the bacterial isolates C3, C4, Vu1, and Vu2 using the 27F and 1492R primers. These isolates showed 95%, 95%, 98%, and 99% similarity with *Brevibacillus parabrevis*, respectively. Based on the 16S rRNA sequencing data, bacterial isolates C5, Vf1, and Vf3 showed 100% similarity with the *Bacillus* genus (Table 3). BLAST comparisons revealed that the nucleotide sequences of the analyzed isolate Vf2 have very high homology to *Sphingobium yanoikuyae* KX507143.1 available in the NCBI GenBank, with 100% similarity (Table 3).

### Estimation of spore density, isolation frequency and relative abundance of AM fungal species

**Fig. 3:**
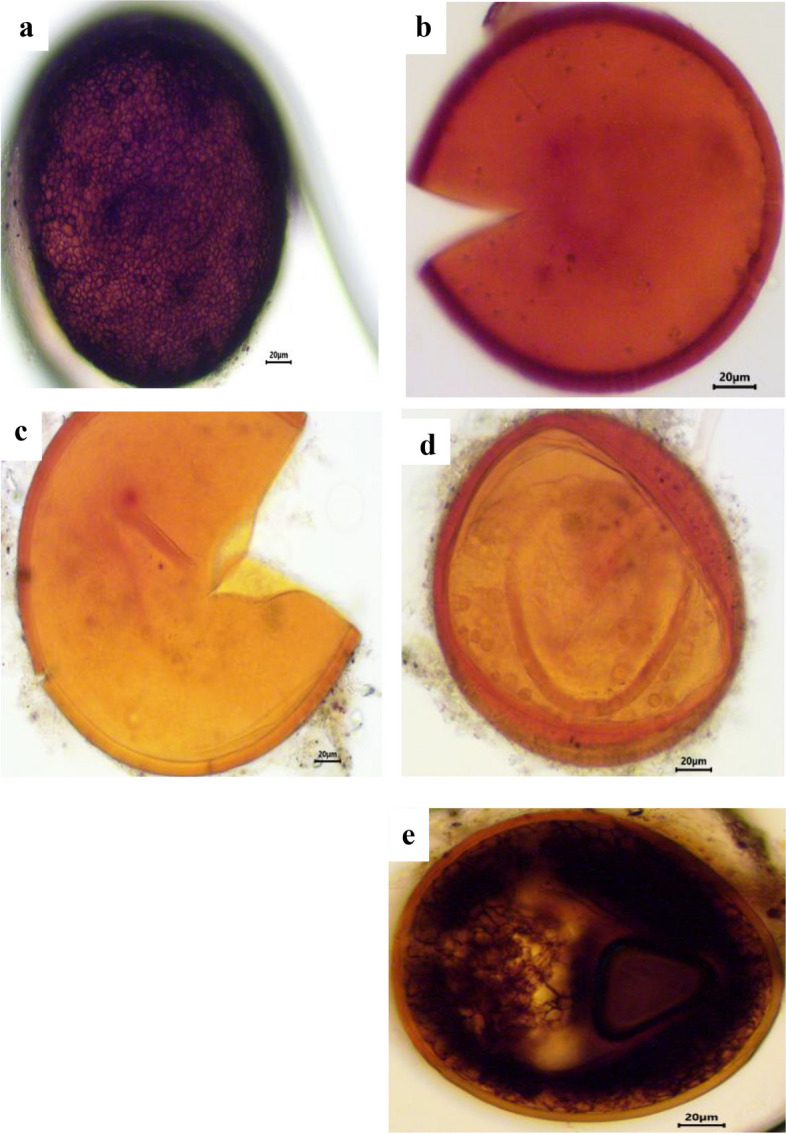
Photomicrographs showing various arbuscular mycorrhizal fungal spores isolated from the rhizosphere soil of chickpea plants. **a** *Acaulospora bireticulata* F.M. Rothwell & Trappe, (**b**) *A. capsicula* Błaszk., (**c**) *A. koskei* Błaszk., (**d**) *A. lacunosa* J.B.Morton. (**e**) *A. thomii* Błaszk

**Fig. 4:**
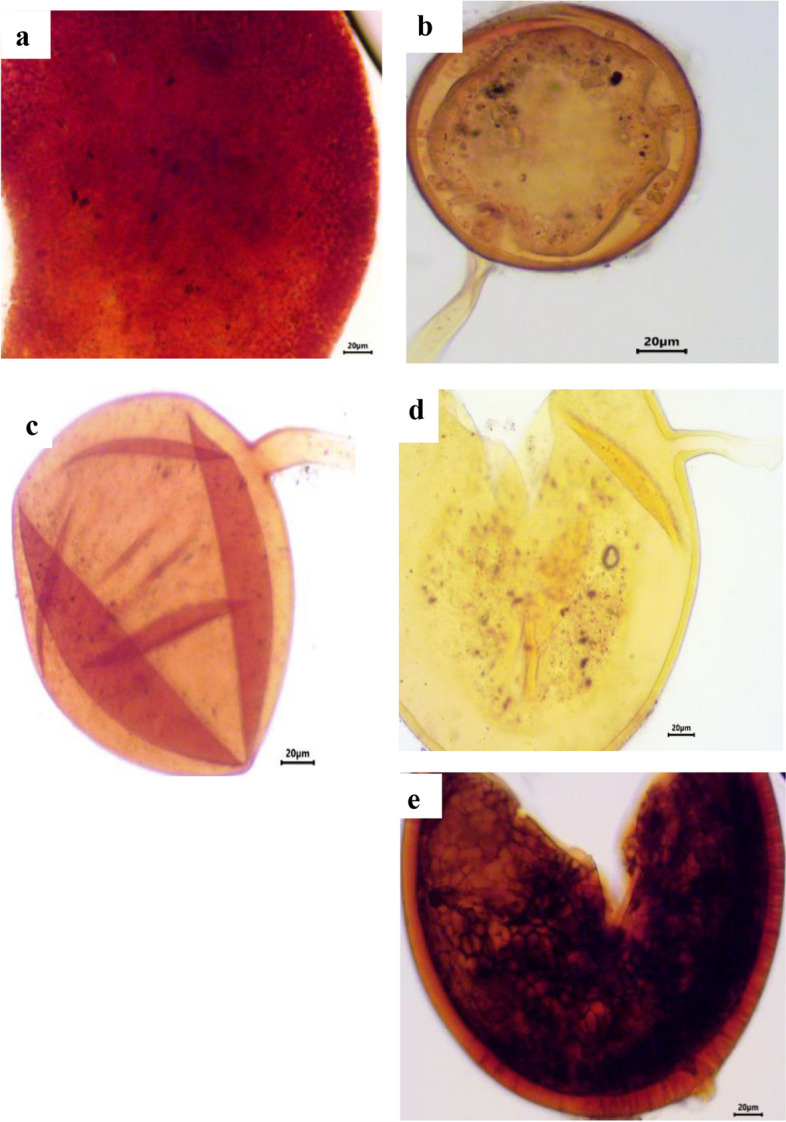
Photomicrographs showing various arbuscular mycorrhizal fungal spores isolated from the rhizosphere soil of chickpea plants. **a** *Ambispora appendicula* (Spain, Sieverd. & N.C. Schenck) C. Walker, (**b**) *Claroideoglomus claroideum* (T.H. Nicolson & Gerd.), (**c**) *Diversispora trimurales* Koske & Halvorson., (**d**) *Funneliformis caledonius* (T.H. Nicolson & Gerd.), (**e**) *F. constrictus* Trappe

**Fig. 5:**
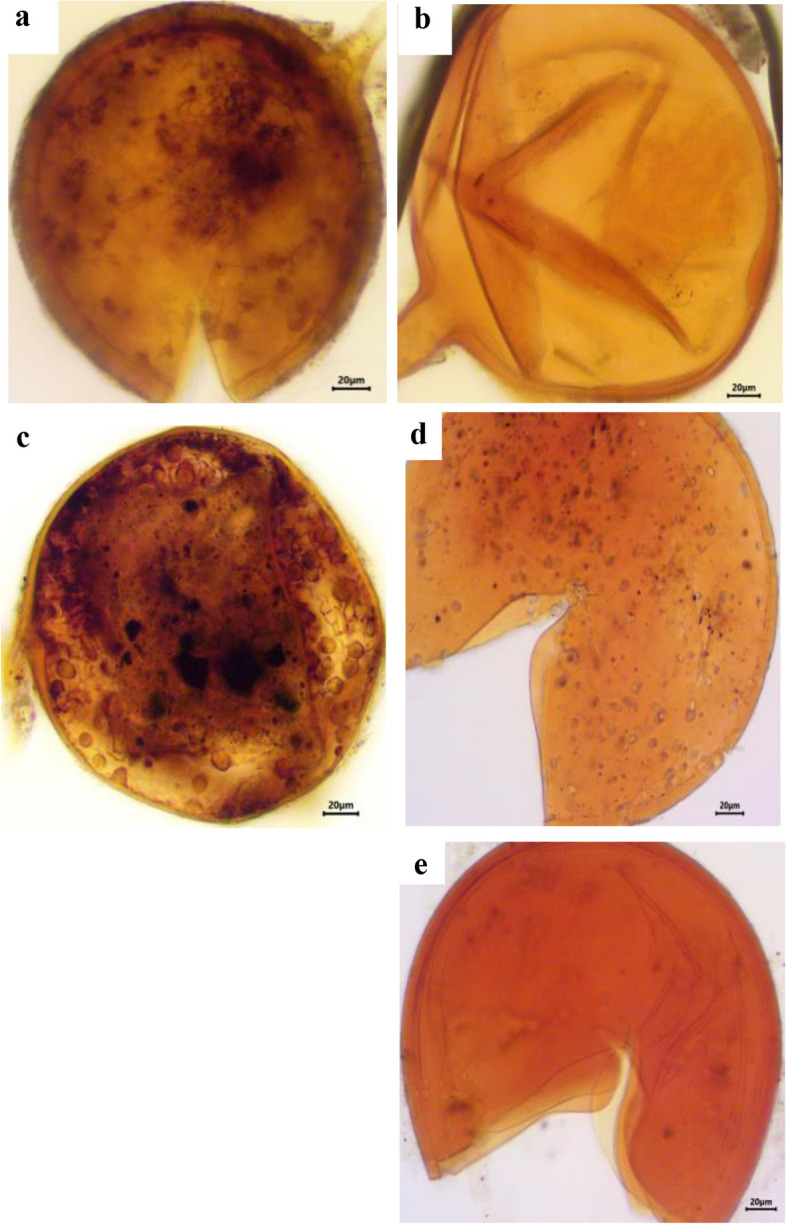
Photomicrographs showing various arbuscular mycorrhizal fungal spores isolated from the rhizosphere soil of.faba bean plants. **a** *F. coronatus* Giovann., (**b**) *F. geosporus* (T.H. Nicolson & Gerd.) C. Walker & A. Schüßler, (**c**) *F. mosseae* (T.H. Nicolson & Gerd.) Gerd. & Trappe. (**d**) *Gigaspora gigantea* (T.H. Nicolson & Gerd.), (**e**) *G. margarita* W.N. Becker & I.R. Hall.,

**Fig. 6:**
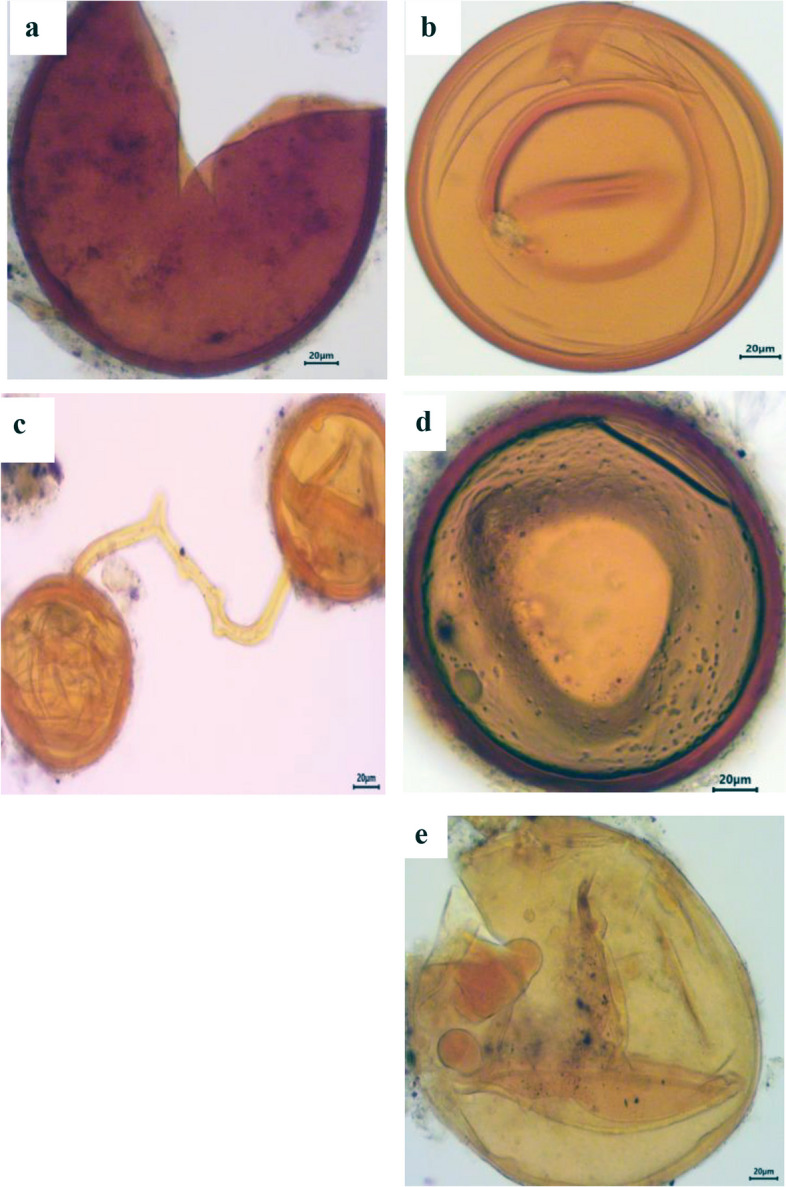
Photomicrographs showing various arbuscular mycorrhizal fungal spores isolated from the rhizosphere soil of faba bean plants. **a** *Glomus caesaris* Sieverd. & Oehl, (**b**) *Pacispora robiginia* Sieverd. & Oehl, (**c**) *Rhizophagus aggregatus* (N.C. Schenck & G.S. Sm.) C. Walker, (**d**) *Scutellospora armeniaca* Błaszk., and (**e**) *S. calospora* (T.H. Nicolson & Gerd.) C. Walker

A total of 37 different species were morphologically identified from the four selected sites in Assiut Governorate. *Acaulospora koskei* and *Acaulospora capsicula* had the highest spore density per 100 g of soil in chickpea (Figs. 3 and 4) and faba bean plants (Figs. 5 and 6). For chickpea plants, the total number of spores and sporocarps of AMF collected from El Qossia, Manfalout, Al Hawatkah, and the Botanical Garden of Faculty of Science were 126, 113, 141, and 150, respectively. For faba bean plants, the total number of spores and sporocarps of AMF collected from the same sites were 206, 211, 247, and 290, respectively.

**Fig. 7:**
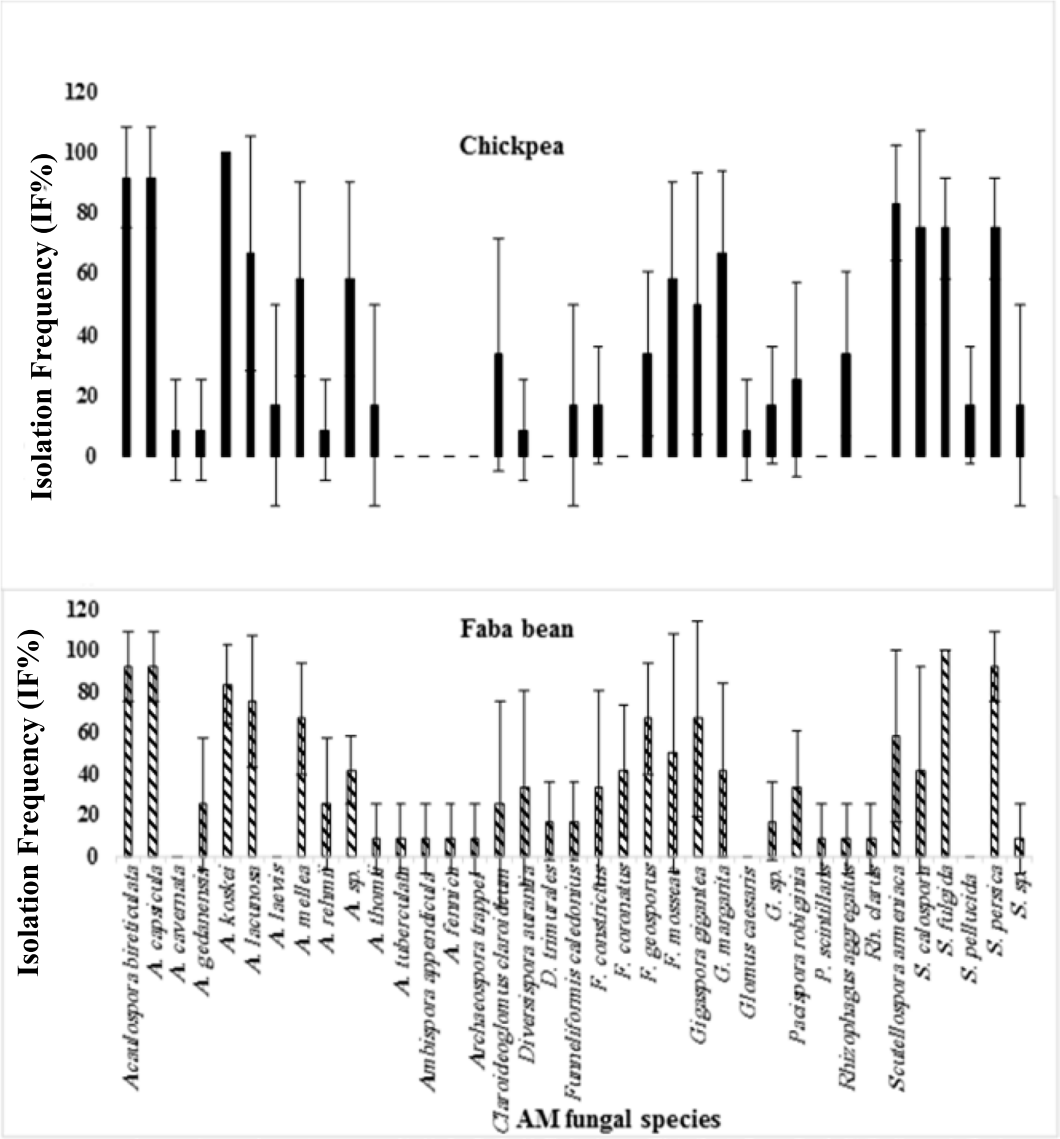
Isolation frequency (IF %) of arbuscular mycorrhizal fungi (AMF) in the fields cultivated with chickpea and faba bean plants

The isolation frequency of each species is depicted in Fig. 7. *Acaulospora koskei* was found to have the highest isolation frequency (100%) in chickpea plants at all selected sites, while *Acaulospora bireticulata, A. capsicula,* and *Scutellospora persica* had the highest isolation frequency (91.66%) in faba bean plants. It is interesting to note that the genus *Archaeospora* is not affected by chickpea plants, and the genus Glomus is not affected by faba bean plants. The relative abundance (RA) of the genera and species of AM fungi is presented in Table 4. In chickpea plants, thirteen species were dominant (RA>2.2%). The most abundant species were *Acaulospora koskei* (20.38%), followed *by Acaulospora capsicula* (10.75%), and then *Acaulospora bireticulata* (10.19%). The family Acaulosporaceae had the highest number of spores in relative abundance. In faba bean plants, eleven species were dominant (RA>2.2%). The most abundant species was *Acaulospora koskei* (17.18%), followed by *Acaulospora capsicula* (11.9%), and then *Scutellospora persica* (10.4%). The family Acaulosporaceae had the highest number of spores in relative abundance, followed by Gigasporaceae.

**Table 4:** Relative abundance (RA %) of arbuscular mycorrhizal fungi (AMF) in the fields cultivated with chickpea and faba bean plants

Arbuscular mycorrhizal fungi	Chickpea	Faba bean
*Acaulospora bireticulata* F.M. Rothwell & Trappe	10.19	8.85
*A. capsicula* Błaszk	10.75	11.9
*A. cavernata* Błaszk	0.19	0
*A. gedanensis* Błaszk	0.19	1.46
*A. koskei* Błaszk	20.38	17.18
*A. lacunosa* J.B.Morton	3.02	3.18
*A. laevis* Gerd. & Trappe	0.38	0
*A. mellea* Spain & N.C. Schenck	2.64	4.29
*A. rehmii* Sieverd. & S.Toro	0.38	1.29
*A.* sp.	1.69	1.2
*A. thomii* Błaszk	0.75	0.86
*A. tuberculata* Janos & Trappe	0	0.52
*Ambispora appendicula* (Spain, Sieverd. & N.C. Schenck) C. Walker	0	0.17
*A. fennica* C. Walker, Vestberg & A. Schüßler	0	0.17
*Archaeospora trappei* (R.N. Ames & Linderman) J.B. Morton & D. Redecker	0	0.17
*Claroideoglomus claroideum* (T.H. Nicolson & Gerd.)	1.13	0.52
*Diversispora aurantia* Błaszk., Blanke, Renker & Buscot	0.19	0.52
*D. trimurales* Koske & Halvorson	0	0.6
*Funneliformis caledonius* (T.H. Nicolson & Gerd.) Trappe & Gerd	0.19	0.69
*F. constrictus* (Trappe) C. Walker & A. Schüßler	1.32	0.52
*F. coronatus* Giovann	0	1.46
*F. geosporus* (T.H. Nicolson & Gerd.) C. Walker	2.64	3
*F. mosseae* (T.H. Nicolson & Gerd.) Gerd. & Trappe	3.58	1.2
*Gigaspora gigantea* (T.H. Nicolson & Gerd.)	2.83	3.87
*G. margarita* W.N. Becker & I.R	3.96	1.29
*Glomus caesaris* Sieverd. & Oehl	0.57	0
*G.* sp.	0.57	0.95
*Pacispora robiginia* Sieverd. & Oehl	0.75	0.95
*P. scintillans* (S.L. Rose & Trappe) Sieverd. & Oehl	0	0.09
*Rhizophagus aggregatus* (N.C. Schenck & G.S. Sm.) C. Walker	1.32	0.34
*Rh. clarus* (T.H. Nicolson & N.C. Schenck) C. Walker & A. Schüßler	0	0.43
*Scutellospora armeniaca* Błaszk	7.17	6.36
*S. calospora* (T.H. Nicolson & Gerd.) C. Walker	5.66	4.29
*S. fulgida* Koske & C. Walker	7.55	8.77
*S. pellucida* (T.H. Nicolson & N.C. Schenck) C. Walker & F.E. Sanders	0.75	0
*S. persica* (Koske & C. Walker) C. Walker	7.74	10.39
*S.* sp*.*	0.19	0.17

### Assessment of mycorrhizal colonization in plant roots

**Fig. 8:**
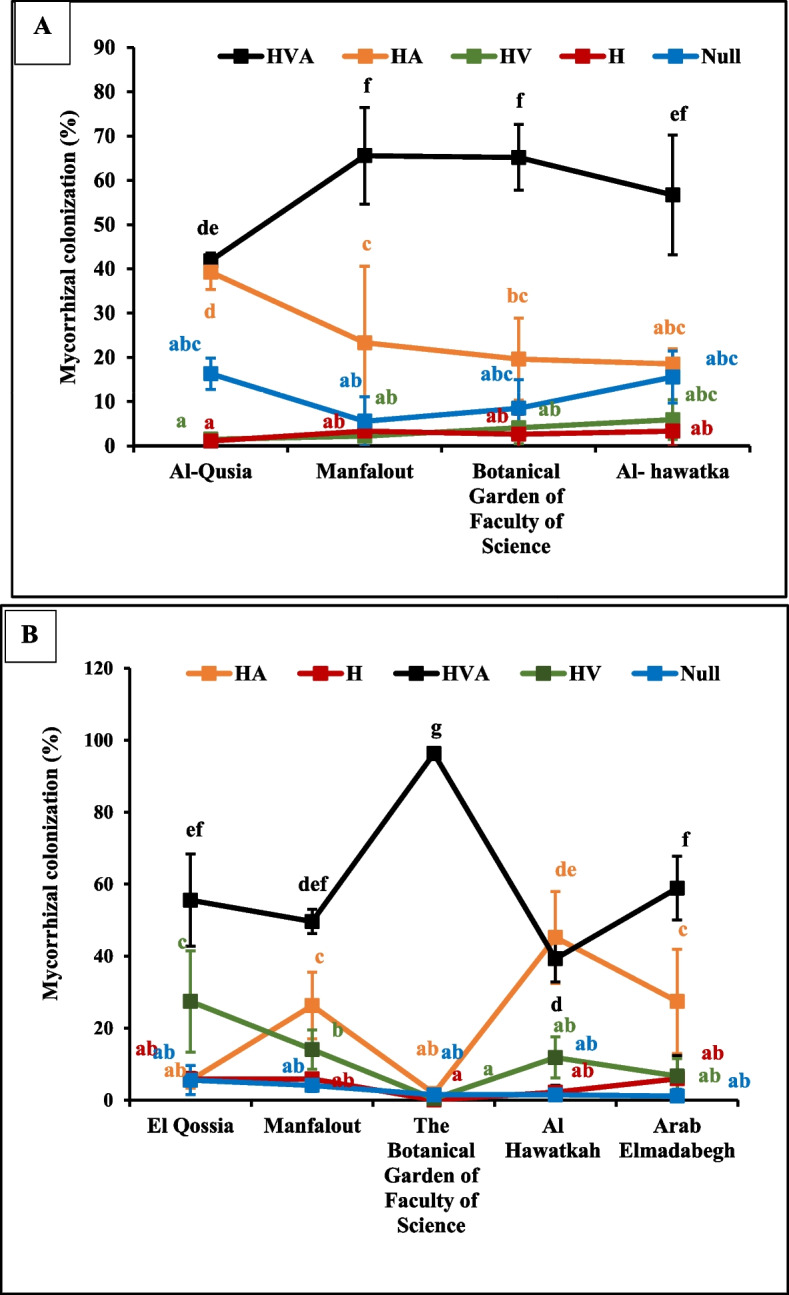
Average percentages of AMF colonization in chickpea (**A**) and faba bean (**B**) roots sampled from various habitats. Hypha, vesicles and arbscules (HVA), Hypha and arbscules (HA), Hypha and vesicles (HV), and Hypha (H). Means followed by different letters are significantly different according to "Duncan's multiple range" test in SPSS

The mycorrhizal colonization refers to the degree of root occupation by mycorrhizal fungi. In this study, the two leguminous plants, *Cicer arietinum* L. and *Vicia faba* L., showed high mycorrhizal root colonization. The colonization of the roots by arbuscular mycorrhizal fungi (AMF) was represented by the presence of internal hyphae, vesicles, and arbuscules. Overall, the root colonization by hyphae, vesicles, and arbuscules (HVA) was highest in different locations in Assiut Governorate (Fig. 8).

## Discussion

Root nodules of leguminous plants host a diverse population of endophytic bacteria, known as non-rhizobial nitrogen-fixing bacteria [3, 4, 50]. In this study, we isolated thirty-four strains from surface-sterilized root nodules of *Cicer arietinum* L., *Vicia faba* L., and* Vigna unguiculata* (L.) Walp. Out of these, thirty-one strains did not induce nodulation. Stajkovic [51] isolated approximately 15 endophytic bacterial strains but could not induce nodulation in alfalfa plants. The nodulation test is used to identify rhizobial strains. The negative results of the nodulation test indicate that these bacterial strains are non-rhizobial and live in the root nodules as endophytic bacteria. Most of these endophytic bacteria were able to utilize various carbon sources such as fructose, galactose, glucose, maltose, sucrose, and mannitol. *Rhizobium* strains were more efficient in utilizing glucose and sucrose compared to normal YEM medium [52, 53]

Ten representative strains were selected for 16S rRNA analysis. These strains belonged to the Proteobacteria and Firmicutes phyla, with five different genera: *Stenotrophomonas*, *Brevibacillus*, *Massilia*,* Bacillus*,* and Sphingobium*. Brígido group isolated endophytic bacteria from root nodules of chickpea plants, including *Stenotrophomonas, Bacillus, Pseudomonas,* and *Enterobacte* [54]. Zakhia [55] reported endophytic bacteria isolated from root nodules collected from spontaneous legumes in the arid zone of Tunisia, belonging to the genera *Agromyces, Bacillus, Microbacterium, Ochrobactrum, Ornithinicoccus, Paenibacillus, Paracraurococcus, Phyllobacterium, Pseudomonas, Rhodopseudomonas,* and *Sphingomonas.* Boukhatem [56] isolated nodular endophytes from root nodules of native Acacia spp., belonging to nine genera: *Paenibacillus, Ochrobactrum, Stenotrophomonas, Pseudomonas, Microbacterium, Rhizobium, Agrobacterium, Brevibacillus,* and *Advenella*. Under saline stress, the endophytic root nodule *Stenotrophomonas maltophilia* (KY515467.1) has been shown to alleviate the damaging effects of salinity on chickpea plants by enhancing nodule formation and biological nitrogen fixation [41]. Marques [57] indicated that the rhizosphere affects certain bacterial taxa, including *Sphingobium* (family Sphingomonadaceae), in field-grown sweet potato. Additionally, Yin group [58] reported that the distribution of the Sphingomonadaceae family is influenced by root proximity, tillage treatment, and location. The endophytic bacteria in root nodules of alfalfa plants (*Medicago sativa* L.) belong to three different genera: *Bacillus, Microbacterium,* and *Brevibacillus * [51]*. Bacillus* species have been found as nodule endophytes in soybean [59], red clover [26], Kudzu (*Pueraria thunbergiana*) [60], *Calycotome villosa* [55], and various wild legumes [50]. The spore-forming capability of many Bacilli is a reason for their adaptation in the field [61].

In this study, most of the recovered isolates are diazotrophic bacteria with plant growth-promoting properties. Non-symbiotic nitrogen-fixing bacteria play a crucial role in creating a healthy environment for legumes in their early growth stages. While legumes primarily depend on symbiotic relationships with rhizobia for nodule formation, non-symbiotic bacteria enrich the soil with bioavailable nitrogen, boosting nitrogen availability for young legume plants and promoting root development and overall plant health [62, 63]. Healthy soil conditions established by non-symbiotic bacteria support the subsequent colonization by rhizobia, facilitating effective nodule formation as the plants mature [64]. Although non-symbiotic nitrogen fixers do not directly induce nodule formation, they significantly contribute to the growth and establishment of legumes during their early developmental phases.

These properties include nitrogen fixation, production of indole acetic acid (IAA), ammonia (NH3), phosphate solubilization, and exopolysaccharide (EPS) production, which are known mechanisms for stimulating plant growth. The production of indole-3-acetic acid (IAA) by bacteria is closely linked to nodule formation in legumes. IAA is a key plant hormone that influences various growth processes, including root development and differentiation [65]. Certain bacteria, such as symbiotic rhizobia and non-symbiotic nitrogen-fixing bacteria, can synthesize IAA, which enhances the root architecture of legumes, promoting better colonization and nutrient uptake [66]. IAA production in the rhizosphere stimulates the growth of lateral roots and root hairs, creating a favorable environment for nodule establishment. This hormone not only aids in root development but also regulates signaling pathways that facilitate the interaction between legume plants and their symbiotic partners [67]. Improved root growth increases contact between plant roots and nitrogen-fixing bacteria, promoting effective nodule formation. Additionally, IAA can enhance plant responses to environmental stresses, improving overall plant health and nodulation [68].

Studies suggest that bacteria producing higher levels of IAA lead to more robust nodule development, underscoring the importance of bacterial IAA production in successful symbiosis between legumes and nitrogen-fixing partners [69–71]. The presence of these endophytic non-rhizobial bacterial strains suggests their potential role in promoting plant growth. In a significant study by Zhao [72], endophytes MQ23 and MQ23R were isolated from the root nodules of *Sophora alopecuroides*, demonstrating their potential to promote plant growth. These endophytes were found to enhance plant growth through various mechanisms, with the production of indole acetic acid (IAA) playing a crucial role in their beneficial interaction with plants [73].

In our study, we assessed the IAA production of the isolated strains. Strain C1, isolated from chickpea, exhibited the highest IAA production, reaching a concentration of 8.94 mg ml^−1^. In contrast, strain C10 showed lower IAA production, measuring at 2.68 mg ml^−1^ compared to other strains. It is important to note that bacterial IAA production is influenced by the presence of tryptophan, which can be obtained from seeds or root exudates [73]. These results highlight the strain-specific differences in IAA production and emphasize the potential of strain C1 as a strong producer of IAA, contributing to plant growth promotion. The ability of these endophytic bacteria to produce IAA, combined with their presence in the root nodules, underscores their importance in facilitating plant-bacteria interactions and influencing plant growth. These findings offer valuable insights into the mechanisms behind the positive effects of these bacteria on plant development and present new opportunities for utilizing their potential in agriculture.

The study's findings highlight the successful screening of bacterial isolates for phosphate solubilization using the Pikovskaya phosphate (PVK) solid medium. Notably, the endophytic bacterial isolate (Vf1) identified as *Bacillus cereus* (MG515188.1) demonstrated a remarkable ability to solubilize phosphate. This finding is consistent with the research conducted by Pandya [61], who isolated *Bacillus anthracis* (M1) from the root nodules of *Vigna radiata* and observed its proficiency in various plant growth-promoting (PGP) traits, including nitrogen fixation, the production of indole acetic acid (IAA) and siderophores, antifungal activity, and phosphate solubilization. Additionally, Idriss [74] reported the phosphate solubilization capability of *Bacillus mucilaginous*. Strains from the genera *Pseudomonas, Bacillus,* and* Rhizobium* are among the most effective phosphate solubilizers [75].

Plant growth-promoting bacteria (PGPB) have the ability to produce ammonia, which is important for the conversion of organic nitrogen into ammonium form, thus enhancing soil nitrogen content [76]. In our study, all bacterial strains except for the C13 isolate demonstrated the capacity to produce ammonia. This aligns with previous research that reported high ammonia production capabilities among the majority of isolates. Additionally, other studies have found that bacterial isolates from the genera *Bacillus, Enterobacter,* and* Pseudomonas* also exhibit the ability to produce ammonia [54]. The production of ammonia by PGPB contributes to nutrient cycling and availability in the soil, serving as a vital source of nitrogen for plants [77]. Our findings confirm the widespread occurrence of ammonia-producing bacterial strains, particularly within the genera *Bacillus, Enterobacter,* and* Pseudomonas*. By actively engaging in ammonification, these bacteria play a crucial role in enriching the soil with accessible nitrogen, ultimately benefiting plant health and productivity.

Exopolysaccharides (EPS) play a crucial role in the complex interactions between endophytes and plants, influencing plant health and development. These complex molecules create a favorable microenvironment for bacterial survival within the plant host and act as a protective barrier, shielding bacteria from the plant's defense mechanisms [78]. An excellent example of a plant growth-promoting rhizobacterium is *Bacillus amyloliquefaciens* FZB42, which not only enhances plant growth but also stimulates resistance to pathogens and increases tolerance to salt stress [79]. This rhizobacterium demonstrates the positive impact of plant–microbe interactions on growth promotion and stress mitigation, highlighting the potential of such relationships in agriculture. In our study of nodule endophytic strains, we found intriguing results regarding *Stenotrophomonas maltophilia* (C1) and *Brevibacillus parabrevis* (Vu2), isolated from the root nodules of *Cicer arietinum* and* Vigna unguiculata,* respectively. These strains demonstrate an exceptional ability to produce high yields of EPS, surpassing previously published data [45]. The significant EPS production by these endophytic strains underscores their potential to establish robust interactions with host plants, suggesting their involvement in promoting overall plant growth, development, and resilience. The profound impact of EPS cannot be underestimated, as these molecules play a critical role in endophyte-plant interactions by creating a favorable microenvironment, enhancing bacterial survival, and acting as a barrier against plant defense mechanisms. These findings contribute to our understanding of EPS-mediated endophyte-plant interactions and offer promising avenues for harnessing the potential of such strains in agriculture, including promoting plant growth and conferring tolerance to various stresses.

Arbuscular mycorrhizal fungi (AMF) are important components of rhizosphere microbial communities in agricultural ecosystems [80]. They are beneficial microbes that play a fundamental role in soil fertility by increasing resistance to environmental stresses, enhancing plant nutrient acquisition, water relations, disease resistance, efficient nutrient recycling, and long-term soil stability [36]. This study aims to describe the biodiversity of AMF in chickpea and faba bean plants cultivated in different locations within Assiut Governorate (El Qossia, Manfalout, Arab Elmadabegh, Al Hawatkah, and the Botanical Garden of the Faculty of Science). A total of thirty-seven species were identified based on microscopic characteristics from these various habitats.

Studies on AMF diversity, based on morphological data, have been conducted in agroecosystems in Europe, India, China, and Africa, with recorded species ranging from 12 to 58 in soil samples [81–83]. In relatively small regions like the Upper Rhine Valley in Germany, France, and Switzerland, the number of species can reach up to 60–70 [84]. In this study, the most dominant species with the highest spore density in most localities cultivated with chickpea was *Acaulospora.* This is a common occurrence as *Acaulospora* is adapted to various environmental conditions [85, 86]. Due to their small spore size, rapid growth, and wide geographical distribution, *Acaulospora* is more easily propagated and likely to survive in disturbed systems [85]. Another study by Khallaf [87] recently identified a total of 83 morphotypes of AMF from wheat fields (45 soil samples) in Assiut Governorate, with 51 species identified as known species. The dominant genus was Glomus, followed by *Acaulospora*. The relative abundance of AMF taxa in the soil depends on the availability of suitable habitat and favorable host plants. Soil factors can also influence the host's response to colonization by an AMF species [88]. The morphology of vegetation hyphae cannot efficiently and precisely be used to differentiate between AM fungal species; however, their spores are discrete units. Spores have complex and conserved morphological traits that are developmentally controlled, enabling their classification into species [89, 90]. Therefore, unlike plants, the reproductive phase of AMF is used to quantify diversity. The findings of the present study indicate that the colonization and relative abundance of plant and fungal species were not constant at all sampling localities. The relationship between sporulation and colonization by AMF varies with different species, as well as the host and soil nutrient levels [91, 92]. In the current results, it is clear that the number of AMF spores in rhizosphere soils differed among plant species of the same habitat. This suggests that AMF distribution does not coincide with the zonation pattern of vegetation. These differences may be related to the different behavior of each AMF species, even in similar ecosystems [93]. A total of 530 spores and sporocarps of AMF were identified from 12 localities cultivated with chickpea plants. These species belonged to six families: Acaulosporaceae, Claroideoglomeraceae, Diversisporaceae, Gigasporaceae, Glomeraceae, and Pacisporaceae. Additionally, 954 spores and sporocarps of AMF were wet sieved from rhizospheric soil samples collected from the same localities cultivated with faba bean plants. These species belonged to the families Acaulosporaceae, Ambisporaceae, Archaeosporaceae, Claroideoglomeraceae, Diversisporaceae, Gigasporaceae, Glomeraceae, and Pacisporaceae. Based on the isolation frequency (IF %), *Acaulospora koskei* was the most widely distributed species for chickpea (100%) at all selected sites, whereas *Acaulospora bireticulata, A. capsicula,* and *Scutellospora persica* had the highest isolation frequency (91.66%) in faba bean plants. A study in Assiut governorate found twenty-six species of arbuscular mycorrhizal fungi (AMF) in thirty cultivated soils [94]. It was reported that *Glomus mosseae* was the most widely distributed species, appearing in 28 out of 30 soil samples (93%). A previous study identified nine morphotypes of arbuscular mycorrhizal fungi (AMF) in soil samples from six locations near the superphosphate factory in Assiut [95]. It was found that *Funneliformis mosseae* was the most prevalent species, present in ten locations with an 83% occurrence frequency, while *Acaulospora koskei* and *Acaulospora mellea* were found in eight locations with a 67% occurrence frequency [96]. It was noted that mycorrhizal spores are concentrated in the top 10 cm of soil [97], with the highest spore density at a depth of 10–20 cm [98].

This study emphasizes the importance of endophytic bacteria and arbuscular mycorrhizal (AM) fungi in enhancing agroecosystem productivity. Several growth-promoting bacterial strains were isolated from the root nodules of *Cicer arietinum* L., *Vicia faba* L., and *Vigna unguiculata* L. These non-rhizobial bacteria can produce plant growth-promoting regulators like indole-3-acetic acid (IAA), ammonia, phosphate-solubilizing compounds, and extracellular polymeric substances (EPS). These factors potentially explain their ability to enhance plant growth. The diversity of AMF in different locations in Assiut Governorate, where chickpea and faba bean are cultivated, was determined using spore density (SD), relative abundance (RA), isolation frequency (IF), and colonization rate (hyphae, vesicles, and arbuscules). Utilizing these endophytes and plants can significantly contribute to sustainable agriculture by reducing reliance on chemical fertilizers and enhancing crop productivity and resilience in changing environmental conditions. These strains can be used as biofertilizers alone or in combination with rhizobial strains, and they can also be utilized with various legumes or non-leguminous plants. The presence of plant growth-promoting (PGP) traits in endophytes holds promise for their potential use as bioinoculants. However, it is important to recognize that their successful application depends on achieving a harmonious balance with the resident plant microbiome [99]. The resident microbiome plays a crucial role in shaping plant health and productivity, and any introduction of exogenous endophytes must consider the existing microbial community dynamics within the plant. The compatibility and interaction between introduced endophytes and the resident microbiome are crucial factors that determine the effectiveness and long-term stability of the bioinoculation strategy [100]. Careful selection and evaluation of endophytes that can integrate and coexist synergistically with the plant's native microbiome are critical for maximizing the benefits of bioinoculation in agricultural systems. When introducing new inoculated endophytes, it is important to consider the complex dynamics of the plant microbiome, which consists of a diverse community of microorganisms interacting with the host plant. When introducing new inoculated endophytes, it is important to consider the complex dynamics of the plant microbiome, which consists of a diverse community of microorganisms interacting with the host plant [101–103]. Establishing a balanced and mutually beneficial relationship between the introduced endophytes and the resident microbiome is crucial for optimal plant growth and development. This balance ensures that the introduced endophytes do not disrupt the existing microbiome but instead contribute synergistically to overall plant health. Therefore, when developing bioinoculants, it is important to carefully select endophytes with compatible traits that can seamlessly integrate into the resident plant microbiome, fostering a harmonious and symbiotic relationship that enhances plant growth and nutrient uptake.

In conclusion, non-rhizobial bacteria and AM fungi isolates recovered from root nodules offer numerous advantages and show great potential for developing inoculant formulations. They possess various plant growth-promoting traits, such as producing phytohormones and enzymes that aid in nutrient acquisition and utilization. Some isolates can also solubilize phosphates, enhance nutrient availability, and suppress plant pathogens by producing antimicrobial compounds. With their plant growth-promoting characteristics and ability to improve nutrient availability, these isolates are valuable assets in sustainable agriculture and biofertilizer production. Further research and development in this area can lead to the effective and environmentally friendly use of these isolates as biofertilizers in different cropping systems.

## Data Availability

The nucleotide sequence of bacterial isolates (C1 to C5), Vf1, Vf2, Vf3, Vu1, and Vu2 isolated from chickpea (*Cicer arietinum* L), faba bean (*Vicia faba*), and cowpea (*Vigna unguiculata* L. Walp) plants, were deposited in the GenBank nucleotide sequence database under accession number KY515467.1 (https://eur01.safelinks.protection.outlook.com/?url=https%3A%2F%2Fwww.ncbi.nlm.nih.gov%2Fnucleotide%2FKY515467.1%3Freport%3Dgenbank%26log%24%3Dnuclalign%26blast_rank%3D1%26RID%3DM1JC3T6T016&data=05%7C02%7Cmhabdalla%40aun.edu.eg%7Cbac9e600ff2647ff60b208dce7fa6111%7C794544284dbe401ab2ab64a5a7069e2b%7C1%7C0%7C638640309773903022%7CUnknown%7CTWFpbGZsb3d8eyJWIjoiMC4wLjAwMDAiLCJQIjoiV2luMzIiLCJBTiI6Ik1haWwiLCJXVCI6Mn0%3D%7C0%7C%7C%7C&sdata=mr64SjrbyU0k7GxN4Ge0onOvFOYSd9MHZ9VXRk15g8E%3D&reserved=0), MH398502.1(https://eur01.safelinks.protection.outlook.com/?url=https%3A%2F%2Fwww.ncbi.nlm.nih.gov%2Fnucleotide%2FMH398502.1%3Freport%3Dgenbank%26log%24%3Dnuclalign%26blast_rank%3D1%26RID%3DM1JG3D30013&data=05%7C02%7Cmhabdalla%40aun.edu.eg%7Cbac9e600ff2647ff60b208dce7fa6111%7C794544284dbe401ab2ab64a5a7069e2b%7C1%7C0%7C638640309773921795%7CUnknown%7CTWFpbGZsb3d8eyJWIjoiMC4wLjAwMDAiLCJQIjoiV2luMzIiLCJBTiI6Ik1haWwiLCJXVCI6Mn0%3D%7C0%7C%7C%7C&sdata=wvLu0WqBbNUmbk4XOeT%2BBPYFHQf2nErBWnCXaTWxVzI%3D&reserved=0). MH400058.1(https://eur01.safelinks.protection.outlook.com/?url=https%3A%2F%2Fwww.ncbi.nlm.nih.gov%2Fnucleotide%2FMH400058.1%3Freport%3Dgenbank%26log%24%3Dnuclalign%26blast_rank%3D1%26RID%3DM1JM6GKR013&data=05%7C02%7Cmhabdalla%40aun.edu.eg%7Cbac9e600ff2647ff60b208dce7fa6111%7C794544284dbe401ab2ab64a5a7069e2b%7C1%7C0%7C638640309773935166%7CUnknown%7CTWFpbGZsb3d8eyJWIjoiMC4wLjAwMDAiLCJQIjoiV2luMzIiLCJBTiI6Ik1haWwiLCJXVCI6Mn0%3D%7C0%7C%7C%7C&sdata=ndHc%2F4%2BHyouAz2Wm32qcTe8V6N0WPiyzT3gdro9HjKQ%3D&reserved=0). MH398500.1(https://eur01.safelinks.protection.outlook.com/?url=https%3A%2F%2Fwww.ncbi.nlm.nih.gov%2Fnuccore%2FMH398500.1%3Freport%3DGenBank&data=05%7C02%7Cmhabdalla%40aun.edu.eg%7Cbac9e600ff2647ff60b208dce7fa6111%7C794544284dbe401ab2ab64a5a7069e2b%7C1%7C0%7C638640309773948508%7CUnknown%7CTWFpbGZsb3d8eyJWIjoiMC4wLjAwMDAiLCJQIjoiV2luMzIiLCJBTiI6Ik1haWwiLCJXVCI6Mn0%3D%7C0%7C%7C%7C&sdata=2585n%2Bzus6bKaw8hXEDgZJEu34J5JNY%2BVjTKOTdEh24%3D&reserved=0), MG515188.1(https://eur01.safelinks.protection.outlook.com/?url=https%3A%2F%2Fwww.ncbi.nlm.nih.gov%2Fnucleotide%2FMG515188.1%3Freport%3Dgenbank%26log%24%3Dnuclalign%26blast_rank%3D1%26RID%3DM1K872J8016&data=05%7C02%7Cmhabdalla%40aun.edu.eg%7Cbac9e600ff2647ff60b208dce7fa6111%7C794544284dbe401ab2ab64a5a7069e2b%7C1%7C0%7C638640309773963578%7CUnknown%7CTWFpbGZsb3d8eyJWIjoiMC4wLjAwMDAiLCJQIjoiV2luMzIiLCJBTiI6Ik1haWwiLCJXVCI6Mn0%3D%7C0%7C%7C%7C&sdata=d22LDG5BLADbmlQID7nZaVy8NZKKajYz1DgxU9ba5O0%3D&reserved=0), MH398516.1(https://eur01.safelinks.protection.outlook.com/?url=https%3A%2F%2Fwww.ncbi.nlm.nih.gov%2Fnucleotide%2FMH398516.1%3Freport%3Dgenbank%26log%24%3Dnucltop%26blast_rank%3D3%26RID%3DM1KRY82N01N&data=05%7C02%7Cmhabdalla%40aun.edu.eg%7Cbac9e600ff2647ff60b208dce7fa6111%7C794544284dbe401ab2ab64a5a7069e2b%7C1%7C0%7C638640309773977218%7CUnknown%7CTWFpbGZsb3d8eyJWIjoiMC4wLjAwMDAiLCJQIjoiV2luMzIiLCJBTiI6Ik1haWwiLCJXVCI6Mn0%3D%7C0%7C%7C%7C&sdata=cRM362SdL5my09hs6PgCnHt6NBG46ryj3tBipPqWP2I%3D&reserved=0), MH398503.1(https://eur01.safelinks.protection.outlook.com/?url=https%3A%2F%2Fwww.ncbi.nlm.nih.gov%2Fnuccore%2FMH398503.1%3Freport%3DGenBank&data=05%7C02%7Cmhabdalla%40aun.edu.eg%7Cbac9e600ff2647ff60b208dce7fa6111%7C794544284dbe401ab2ab64a5a7069e2b%7C1%7C0%7C638640309773990496%7CUnknown%7CTWFpbGZsb3d8eyJWIjoiMC4wLjAwMDAiLCJQIjoiV2luMzIiLCJBTiI6Ik1haWwiLCJXVCI6Mn0%3D%7C0%7C%7C%7C&sdata=FXOoZ6e14bX7OaPTeF29Q2y%2BVLq%2FEpbmQXWtJVrWBrA%3D&reserved=0), MH398497.1(https://eur01.safelinks.protection.outlook.com/?url=https%3A%2F%2Fwww.ncbi.nlm.nih.gov%2Fnuccore%2FMH398497.1%3Freport%3DGenBank&data=05%7C02%7Cmhabdalla%40aun.edu.eg%7Cbac9e600ff2647ff60b208dce7fa6111%7C794544284dbe401ab2ab64a5a7069e2b%7C1%7C0%7C638640309774003719%7CUnknown%7CTWFpbGZsb3d8eyJWIjoiMC4wLjAwMDAiLCJQIjoiV2luMzIiLCJBTiI6Ik1haWwiLCJXVCI6Mn0%3D%7C0%7C%7C%7C&sdata=hLhT%2F3oov3PQTh0yh4gYJV4A3SglVk4uPvgFp0wHFOE%3D&reserved=0), and. KY515468.1(https://eur01.safelinks.protection.outlook.com/?url=https%3A%2F%2Fwww.ncbi.nlm.nih.gov%2Fnuccore%2FKY515468.1%3Freport%3DGenBank&data=05%7C02%7Cmhabdalla%40aun.edu.eg%7Cbac9e600ff2647ff60b208dce7fa6111%7C794544284dbe401ab2ab64a5a7069e2b%7C1%7C0%7C638640309774016863%7CUnknown%7CTWFpbGZsb3d8eyJWIjoiMC4wLjAwMDAiLCJQIjoiV2luMzIiLCJBTiI6Ik1haWwiLCJXVCI6Mn0%3D%7C0%7C%7C%7C&sdata=OFUdaJCwCC2%2F6u6ro%2Fc8%2BaP13%2BRoSj7qfXTByJ6WCXE%3D&reserved=0), respectively.
